# StrokeCT-2C5K: A Two-Center Cranial CT Dataset for Four-Class Stroke Classification Using SE-Attention-Enhanced Deep Learning Models

**DOI:** 10.3390/biomimetics11090638

**Published:** 2026-09-06

**Authors:** Ahmet Bahadır Karlı, Murat Ucan, Buket Kaya

**Affiliations:** 1 Department of Strategic Information Management Systems, Faculty of Medicine, Dicle University, Diyarbakir 21200, Turkey; ahmet.karli@dicle.edu.tr; 2 Department of Computer Technologies, Vocational School of Technical Sciences, Dicle University, Diyarbakir 21200, Turkey; 3 Department of Electronics and Automation, Firat University, Elazig 23119, Turkey

**Keywords:** StrokeCT-2C5K, stroke classification, cranial computed tomography, deep learning, convolutional neural networks, squeeze and excitation, channel attention

## Abstract

Stroke is one of the leading causes of mortality and long-term neurological disability worldwide, and early diagnosis through accurate disease classification directly affects treatment success. Rapid differentiation of hemorrhagic and ischemic stroke on computed tomography (CT) images, together with accurate determination of the acute and chronic phase in ischemic cases, is of critical importance in the clinical decision-making process. In this study, StrokeCT-2C5K a two-center dataset comprising 5000 cranial CT images, was assembled specifically for this work. The images were reviewed by radiology specialists and assigned to one of four diagnostic categories: normal, hemorrhagic stroke, acute ischemic stroke, or chronic ischemic stroke. A Squeeze-and-Excitation (SE-Attention) mechanism was then integrated into DenseNet-121, ResNet-50, and EfficientNet-B3. From a biomimetic perspective, this channel-recalibration process provides a functional analogy to biological selective attention by giving greater weight to informative responses while reducing the influence of less relevant ones. All models were trained under the same training, validation, and test protocol; the standard CNN architectures were compared with their SE-Attention-enhanced counterparts. The results showed that the SE-Attention mechanism enables more effective learning of lesion-specific discriminative features by adaptively recalibrating channel-wise information, yielding an average classification accuracy improvement of 0.93 percentage points across all three architectures. The most pronounced improvements were observed in distinguishing ischemic from hemorrhagic stroke, as well as in distinguishing acute from chronic ischemic stroke. These findings show that a selective-information-processing strategy functionally analogous to biological attention can improve multi-class stroke classification across different CNN backbones.

## 1. Introduction

Stroke, which occurs when blood flow to a region of the brain is disrupted by occlusion or hemorrhage, is the second leading cause of death worldwide [1]. Early and accurate diagnosis is of great importance for limiting brain damage and improving the chances of recovery. Because treatment decisions in stroke are time-sensitive, deep-learning-based image analysis has been investigated as a means of supporting image interpretation and reducing diagnostic delays [2].

In clinical practice, computed tomography (CT) is the first imaging protocol used, as it provides results much faster than magnetic resonance imaging (MRI) (an average of 1 min versus an average of 8–10 min) and better demonstrates hemorrhage in the early period when hemorrhagic stroke is suspected [3]. However, the approximately ten minutes that a patient with suspected stroke spends in the confined space of an MRI scanner poses a clinical risk.

Stroke is fundamentally divided into two main pathological types, ischemic and hemorrhagic; ischemic stroke results from reduced or halted blood flow, whereas hemorrhagic stroke results from bleeding within brain tissue [4], with ischemic stroke accounting for approximately 87% of all stroke cases [1,3]. Rapid and accurate differentiation between these two types is of critical importance, as it directly determines the intervention to be applied to the patient [3]. However, interpretation of CT images by expert radiologists is both time-consuming and error-prone.

Convolutional neural networks have become a widely used approach in medical image analysis, while vision-transformer-based architectures have more recently emerged as an alternative framework [2,5]. Numerous studies have been conducted in this field in the literature: Chin et al. proposed a CNN-based automated system for the early detection of ischemic stroke [6]. Hossain et al. tested a hybrid ViT-LSTM architecture on CT images using the BrSCTHD-2023 and Kaggle datasets, outperforming conventional CNN architectures [7]. Chen et al. classified 24,769 CT images obtained from 1715 patients into four classes, achieving 98.72% accuracy [8]. Alhatemi and Savaş achieved 98.80% accuracy on MRI images using an EfficientNet-B2 architecture, outperforming other pretrained models [9]. Lee et al. classified five ICH subtypes with performance comparable to expert radiologists using an explainable deep learning system on a small dataset [10]. Chang et al. developed a hybrid 3D/2D convolutional neural network for the detection and quantitative evaluation of intracranial hemorrhage on head CT images [11]. Lo et al. achieved an accuracy of 80–85% in ischemic stroke detection using an AlexNet-based deep CNN model [3].

CT-based deep learning studies have not addressed stroke as a uniform classification problem. Some studies have been designed around a specific diagnostic target, such as the assessment of acute ischemic stroke or the detection of acute intracranial hemorrhage. Other studies have used broader label sets [3]. Chen et al., for example, classified unenhanced brain CT images into normal, acute or subacute hemorrhage, acute infarction, and other findings [8]. Although these studies demonstrate the feasibility of automated CT interpretation, their models address different clinical questions because their diagnostic categories, image sources, and evaluation protocols are not directly comparable. In particular, a system developed only to detect hemorrhage or acute infarction does not necessarily provide a framework for distinguishing acute ischemic changes from chronic ischemic findings.

The composition of the dataset is therefore central to both model development and the interpretation of classification performance. A dataset containing only stroke and non-stroke images can support a binary detection task, whereas a dataset that includes normal findings, hemorrhagic stroke, acute ischemic stroke, and chronic ischemic stroke allows these radiological appearances to be evaluated within a common classification setting. Such a design is also useful when assessing an architectural modification such as channel attention, because an improvement in overall accuracy may result from changes in only one or two classes. Class-specific evaluation is consequently necessary to determine whether the modification contributes to the clinically more difficult distinctions.

From a biomimetic standpoint, the attention component examined in this study relates to the general biological principle of selective information processing. Biological attention does not assign equal importance to every incoming signal; instead, competing inputs are filtered and processing resources are directed toward information that is most relevant to the current task [12]. The SE block implements a limited computational counterpart to this principle by summarizing the responses of convolutional feature channels and assigning greater weight to channels that are more informative for classification [13]. This correspondence is functional rather than anatomical, since the SE block is not intended to reproduce the neural circuitry of human attention. In the present CT classification task, this design allows lesion-related channel responses to be strengthened while less useful responses are reduced. The biomimetic contribution of the study therefore lies in evaluating whether this selective-information-processing principle remains effective when implemented across three structurally different CNN backbones.

In the present study, a two-center cranial CT image dataset was constructed specifically for this purpose. The dataset was not adopted from a pre-existing public benchmark; it was assembled within the scope of the study from images obtained from two institutional radiology archives and organized according to a common four-class labeling scheme. The resulting dataset includes normal CT images, hemorrhagic stroke images, acute ischemic stroke images, and chronic ischemic stroke images. Accordingly, the study had two related objectives: first, to construct a radiologically categorized CT image collection that supports four-class stroke classification; second, to use this dataset to compare DenseNet-121, ResNet-50, and EfficientNet-B3 with their externally SE-augmented counterparts under the same experimental protocol.

## 2. Materials and Methods

### 2.1. StrokeCT-2C5K Dataset

StrokeCT-2C5K, the dataset used in this retrospective observational study, was assembled specifically for the present work. Cranial computed tomography (CT) images were obtained from the radiology archives of Elazığ Fethi Sekin City Hospital and Dicle University Faculty of Medicine Hospital. The name StrokeCT-2C5K denotes a stroke CT image dataset comprising 5000 images collected from two institutional centers. Images from the two institutional sources were brought together to establish a common image collection for model development and evaluation. The dataset was therefore treated as a two-center collection rather than as a combination of previously published public datasets. The study was approved by the Fırat University Social and Human Sciences Research Ethics Committee on 4 November 2025 (session no. 2025/24, decision no. 17), and institutional authorization for the use of anonymized CT images was obtained from Dicle University Hospital on 3 December 2025 (document no. E-22040584-900-1047310).

The images were independently reviewed and labeled by two specialist radiologists with 20 and 4 years of radiological experience. Each reader assigned the images to one of four categories on the basis of their visual CT appearance and professional radiological judgment: normal CT with no radiological evidence of stroke (NS), hemorrhagic stroke (HE), acute ischemic stroke (IA), or chronic ischemic stroke (IK). The distinction between acute and chronic ischemic findings was based on their radiological appearance on CT; a predefined symptom-onset threshold was not applied. Diagnostic labeling was performed by the radiologists before image processing and model development. Following the independent assessments, the labels were jointly reviewed and finalized by consensus. The resulting consensus labels were used as the reference labels throughout model training and evaluation. This four-class structure was intended to represent more than the presence or absence of stroke by separating hemorrhagic and ischemic findings and by treating acute and chronic ischemic appearances as distinct categories.

The final dataset comprised 5000 cranial CT images from 2980 unique patients. Elazığ Fethi Sekin City Hospital contributed 4000 images from 2355 patients: 2000 NS images from 850 patients, 1000 HE images from 750 patients, and 1000 IA images from 755 patients. Dicle University Faculty of Medicine Hospital contributed 1000 IK images from 625 patients. Some patients contributed more than one image, but these images represented different axial slices from a single CT examination; repeat examinations acquired from the same patient at different time points were not included. Overall, NS images accounted for 40% of the dataset, while each stroke-related category accounted for 20%.

At Elazığ Fethi Sekin City Hospital, the CT images were acquired using two Philips Ingenuity CT Core 128 systems (128-slice; Philips Healthcare, Eindhoven, The Netherlands) and one Philips Brilliance CT Big Bore Radiology system (16-slice; Philips Healthcare, Eindhoven, The Netherlands). At Dicle University Hospital, the images were acquired using a Philips Brilliance 64 Channel system (64-slice; Philips Healthcare, Eindhoven, The Netherlands), a Toshiba Activion 16 system (16-slice; Toshiba Medical Systems, Tokyo, Japan), and a GE Revolution EVO CT scanner (64-slice; GE Healthcare, Milwaukee, WI, USA). The archived images were reviewed and labeled using the SECTRA Radiological Imaging and Archiving System (PACS; Sectra AB, Linköping, Sweden).

Before model development, the images obtained from the two institutional archives were standardized to a spatial resolution of 512 × 512 pixels and stored in PNG format with a reported color depth of 32 bits. This standardization established a common image geometry and file format before the model-specific preprocessing stage. The images were subsequently resized to the model input resolution during implementation, as described in the following subsection.

**Figure 1:**
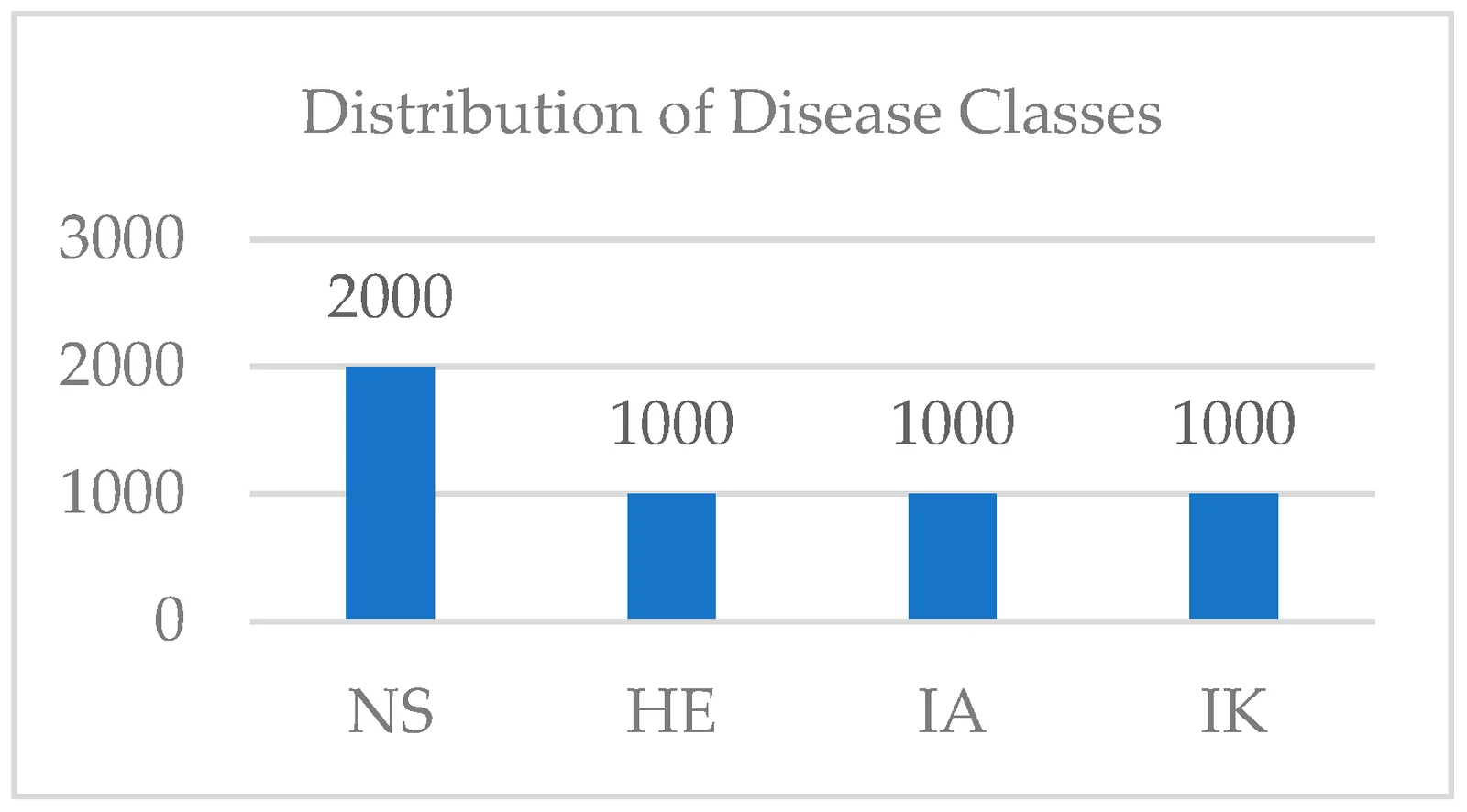
Distribution of diagnostic classes in StrokeCT-2C5K.

**Figure 2:**
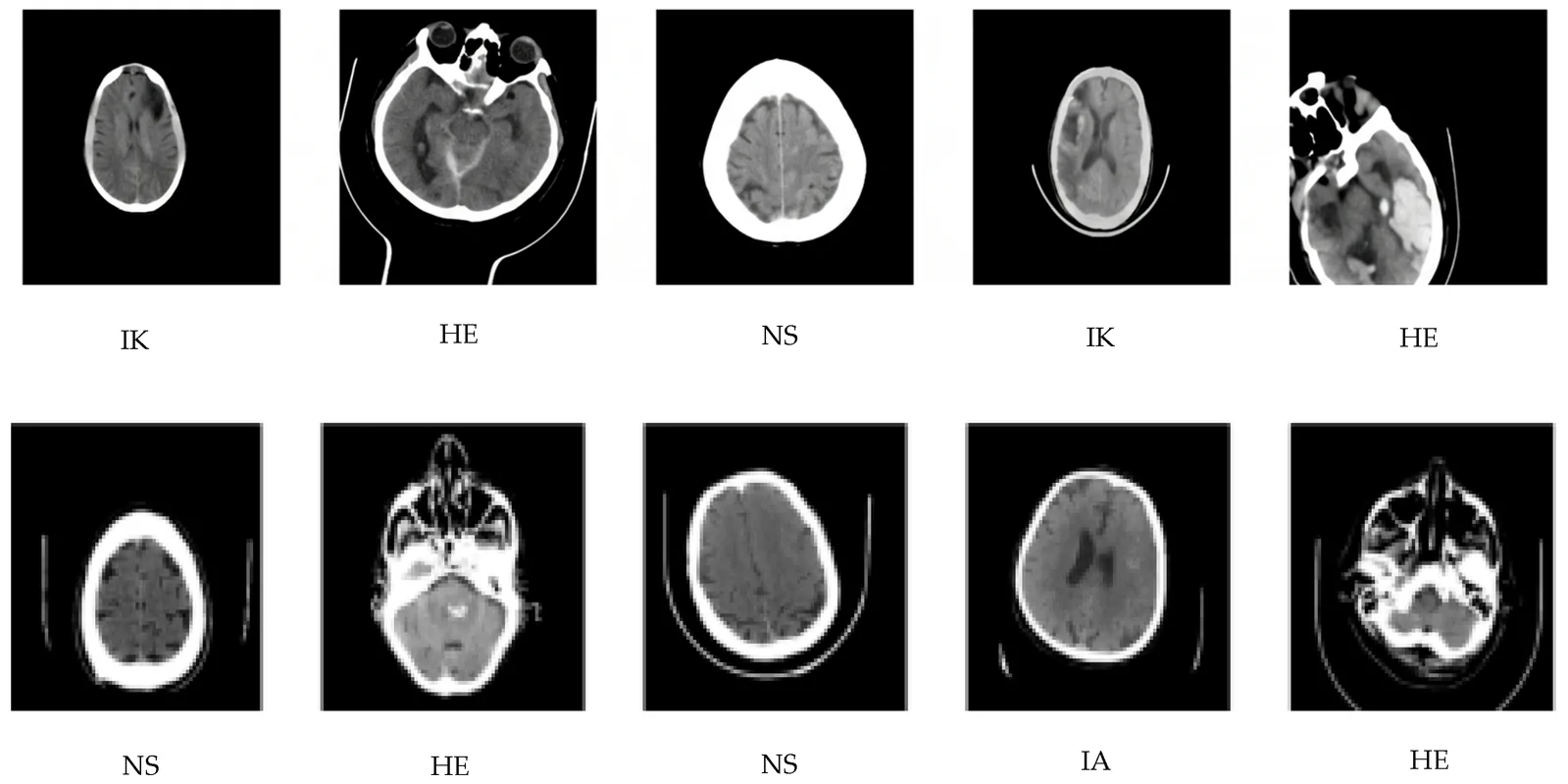
Representative cranial CT images from StrokeCT-2C5K.

The construction of StrokeCT-2C5K and its four-class labeling scheme constitute a central component of the study. The same image collection was used in all experiments so that the baseline architectures and their SE-augmented versions could be assessed under a common data setting. The numerical distribution of the four diagnostic categories is presented in Figure 1, and representative CT images from the dataset are shown in Figure 2.

### 2.2. Backbone Architectures

Backbone selection was treated as a methodological decision because the four diagnostic categories considered in this study do not present the same visual classification problem. Hemorrhagic lesions are generally associated with relatively conspicuous hyperattenuating findings, whereas acute ischemic changes can be more subtle and may involve limited differences in tissue contrast [3]. Chronic ischemic lesions, in turn, are characterized by more established structural changes. Distinguishing these findings from normal cranial CT images requires a model that can preserve fine local information while also constructing higher-level representations of the overall image. Previous studies have shown that CNN-based models can support stroke assessment on brain CT, although their performance is influenced by the selected architecture, data composition, and training strategy [3,8].

DenseNet-121, ResNet-50, and EfficientNet-B3 were selected because they address deep feature learning through different connectivity and scaling strategies [14]. ResNet propagates information through residual addition, DenseNet reuses preceding feature maps through concatenation, and EfficientNet combines compound scaling with mobile inverted bottleneck blocks. The purpose was therefore not to perform an exhaustive comparison of all available CNN architectures. Instead, three established but structurally distinct backbones were evaluated under the same experimental conditions. Using identical images, data partitions, preprocessing procedures, training duration, and external SE-block placement makes it possible to examine whether the contribution of channel recalibration is consistent across architectures or depends on the way each backbone constructs its feature representation. The relevance of such a comparison has also been demonstrated in stroke imaging, where differences among CNN architectures were found to affect both classification performance and external generalization on CT angiography [14].

EfficientNet-B3 was included as the scaled and computationally oriented member of the comparison. The EfficientNet family differs from architectures that increase network depth, width, or input resolution independently. Its compound-scaling strategy adjusts these dimensions together, while MBConv blocks combine inverted bottlenecks and depthwise convolution to control computational demand [15,16]. The practical value of EfficientNet-B3 lies in the balance it offers between representational capacity and resource use, rather than in being uniformly smaller than every alternative architecture. In the present study, the input size was kept at 256 × 256 pixels, as it was for the other backbones, so that input resolution would not become an additional source of variation [17]. EfficientNet-B3 also contains SE operations within its MBConv blocks. Consequently, the external SE block evaluated here represents an additional channel-recalibration stage rather than the first use of channel attention in this architecture [18]. This characteristic makes EfficientNet-B3 particularly useful for determining whether a second, terminal recalibration step provides further benefit after internally recalibrated features have already been produced [16,19].

ResNet-50 was selected as a well-established residual backbone and as a reference architecture with a different information-propagation mechanism. Its bottleneck blocks use shortcut connections to add the input of a block to its transformed output. These connections provide a direct route for information and gradients through the network, which helps maintain stable optimization as network depth increases. In cranial CT classification, this structure can support the learning of higher-level representations while retaining information carried through the residual path. ResNet-50 has also been used in previous transfer-learning studies for the classification of hemorrhagic and ischemic stroke on brain CT [8]. Its main disadvantage is that it generally carries a greater parameter and computational burden than more compact alternatives. Moreover, residual addition does not explicitly preserve every preceding feature map in the way dense concatenation does. Because the standard ResNet-50 backbone does not contain an SE mechanism, its comparison with the externally augmented version provides a relatively direct assessment of the contribution of channel recalibration.

DenseNet-121 represents the feature-reuse strategy in the present comparison. Within each dense block, a layer receives the feature maps produced by all preceding layers and passes its own output to the layers that follow. These connections are implemented by concatenation rather than addition. As a result, low-level information such as edges, local intensity transitions, and texture patterns can remain available to deeper layers alongside more abstract features. This property may be useful in cranial CT, particularly when diagnostically relevant changes are small or have limited contrast. Dense connectivity also supports gradient propagation and allows the network to reuse existing features instead of repeatedly relearning similar representations. A comparative study of large-vessel-occlusion detection on CT angiography reported strong external-validation performance for DenseNet-121 relative to several competing CNN backbones, including residual and EfficientNet models [14]. Its parameter efficiency, however, does not eliminate computational limitations: repeated feature-map concatenation can increase memory use and data movement during training. The external SE block was therefore used to determine whether channel-wise reweighting could further refine the combined feature representation produced by the final dense block.

Taken together, the three backbones provide a complementary basis for evaluating the proposed framework. EfficientNet-B3 offers a controlled balance between scaling and computational demand but already contains internal SE operations. ResNet-50 provides stable residual learning and a widely used reference structure, although it has a comparatively larger parameter burden. DenseNet-121 promotes feature reuse and parameter efficiency, while its dense concatenations can increase memory requirements. None of these properties makes one architecture universally preferable. Their inclusion instead allows the external SE block to be examined across three different feature-propagation mechanisms. Since all other experimental conditions were kept unchanged, the resulting differences can be interpreted more clearly as an interaction between external channel recalibration and backbone structure rather than as a consequence of inconsistent preprocessing or training settings.

### 2.3. SE-Augmented Classification Framework and Experimental Protocol

#### 2.3.1. Image Preprocessing, Dataset Partitioning, and Augmentation

Before being provided to the networks, the PNG images were resized from 512 × 512 to 256 × 256 pixels. The images were then divided into training, validation, and test sets at an 80%–10%–10% ratio while preserving the original class proportions. Partitioning was performed at the image level rather than the patient level. Consequently, different axial slices from the same CT examination could have been assigned to different partitions.

The training partition contained 4000 images, including 1600 NS images and 800 images from each of the HE, IA, and IK classes. The validation and test partitions each contained 500 images, comprising 200 NS images and 100 images from each of the three stroke-related classes. The same partitions were used for all baseline and SE-augmented model comparisons.

Training-set balancing was performed after image-level partitioning and was restricted to the training set to provide equal class representation during model optimization. The original training partition contained 1600 NS images and 800 images from each of the HE, IA, and IK classes. To limit the influence of the larger NS class during training, 1000 NS images were randomly retained using a fixed random seed of 123. For each stroke-related class, 200 additional images were generated exclusively from images already assigned to the training partition. This produced a balanced training set containing 1000 images per class and preserved the total training-set size at 4000 images.

Augmentation included random horizontal flipping, rotation between −20° and +20°, horizontal and vertical translation of up to 20% of the image dimensions, and random zooming of up to 20%. In total, 600 augmented images were generated: 200 for HE, 200 for IA, and 200 for IK. Images assigned to the validation and test sets were not used as augmentation sources, and neither set was augmented, downsampled, or resampled. Their original class distributions were retained throughout model development. The image counts resulting from image-level partitioning and the training-set balancing procedure are summarized in Table 1. Horizontal flipping was considered label-preserving for the present classification task because the four diagnostic categories were defined independently of lesion laterality. Although flipping reverses the left–right orientation, lesion side was not used as a prediction target, and the transformation was applied only to the training set.

#### 2.3.2. Integration of the External SE Block

The SE module was implemented as an external channel-recalibration block positioned between the final convolutional feature extractor and the classification head of each backbone. The same placement was used for DenseNet-121, ResNet-50, and EfficientNet-B3, allowing the original feature-extraction structures of the backbones to remain unchanged. This common design also enabled the contribution of the additional attention block to be evaluated under comparable experimental conditions.

Let $F\in R^{H\times W\times C}$ denote the feature tensor produced by the final convolutional layer, where H and W represent the spatial dimensions and C represents the number of feature channels. In the squeeze stage, each channel was summarized by global average pooling:

$$z_{c}=\frac{1}{HW}\sum_{i=1}^{H}\sum_{j=1}^{W}F_{c}\left(i,j\right),\;\;c=1,\ldots ,C$$

The resulting channel descriptor *z* was passed through two fully connected layers. The first layer reduced the channel dimension from C to C/r, whereas the second layer restored it to C. A reduction ratio of r = 16 was used in all experiments. ReLU activation was applied after the first fully connected layer, and sigmoid activation was used to obtain channel-wise attention coefficients:

$$s=\sigma \left(W_{2}\delta \left(W_{1}z\right)\right)$$

$W_{1}\in R^{\left(C/r\right)\times C}$ and $W_{2}\in R^{C\times \left(C/r\right)}$ denote the learnable weight matrices, δ denotes the ReLU function, and σ denotes the sigmoid function. This operation follows the channel-recalibration formulation introduced by Hu et al. [13].

In the scaling stage, each channel of the original feature tensor was multiplied by its corresponding attention coefficient:


$$\tilde{F_{c}}=s_{c}F_{c}$$

The recalibrated feature tensor was subsequently passed to global average pooling and the final fully connected classification layer with softmax activation. The output layer contained four neurons corresponding to normal CT images without stroke findings (NS), hemorrhagic stroke (HE), acute ischemic stroke (IA), and chronic ischemic stroke (IK).

**Figure 3:**
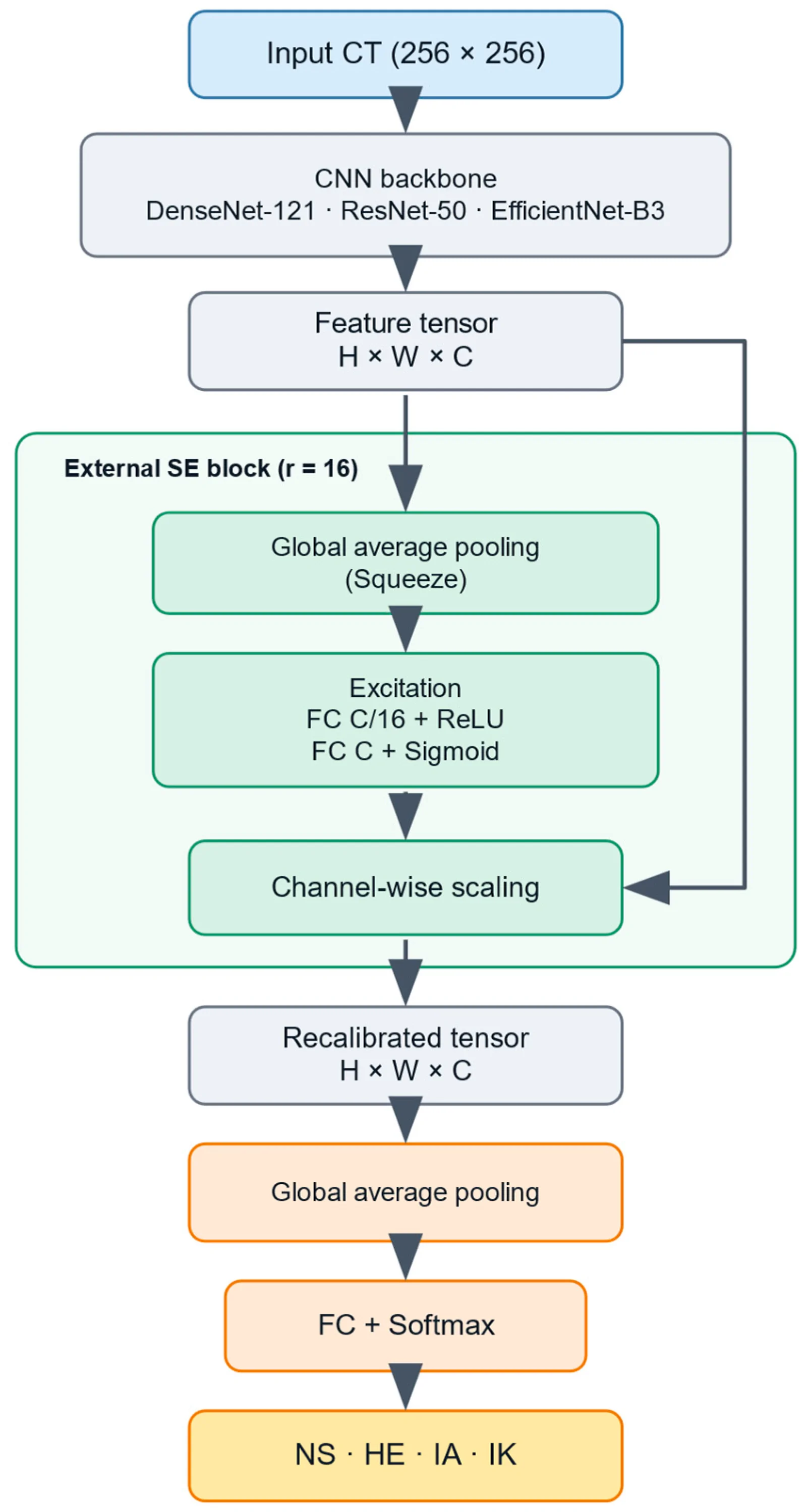
Integration of the external squeeze-and-excitation block into the CNN-based stroke classification framework.

For DenseNet-121 and ResNet-50, the external SE block introduced channel recalibration at the output of the final convolutional feature extractor. EfficientNet-B3 already contains internal SE operations within its MBConv blocks; therefore, the module used in the present study represents an additional external recalibration stage rather than the first introduction of SE operations into this backbone [17]. The external block was placed at the same relative position and the reduction ratio was fixed at r = 16 for all three architectures. Apart from this external block, the corresponding baseline and SE-augmented models were evaluated using the same preprocessing steps, data partitions, and training settings. The position of the external SE block within the classification framework is illustrated in Figure 3.

#### 2.3.3. Training Configuration and Model Evaluation

All experiments were conducted in Python 3.11.4 using TensorFlow/Keras in the Kaggle notebook environment with NVIDIA Tesla T4 GPU. Backbones DenseNet-121, ResNet-50 and EfficientNet-B3 were initialized with ImageNet pre-trained weights and fine-tuned from the first epoch. The newly added SE and classification layers had the default Keras initialization parameters. All configurations were trained for 40 epochs using the Adamax optimizer, categorical cross-entropy loss, batch size of 40 and learning rate of 0.001. A custom callback monitored validation loss during training. At the end of training, the weights corresponding to the lowest validation loss were retained for test evaluation. A random seed of 123 was used for image-level partitioning, NS-class subsampling, and the Dropout layer; no additional global TensorFlow or NumPy seed was set. The reported results were obtained from training run for each configuration under the same fixed data partitions and training settings.

The validation set was used to monitor model performance during training, whereas the held-out test set was reserved for final evaluation. Test performance was reported using overall classification accuracy and class-specific precision, recall, and F1-score. The macro-averaged F1-score was additionally calculated to summarize performance across the four diagnostic classes with equal contribution from each class. Confusion matrices were used to examine the distribution of correct and incorrect predictions among the NS, HE, IA, and IK classes. Training time was recorded for each baseline and SE-augmented model and was reported together with the classification results. Differences between the baseline and SE-enhanced configurations were treated as descriptive performance differences in the fixed test set. Reported improvements are defined within the scope of the study as explanatory performance differences rather than implying statistical superiority.

## 3. Results

The standard DenseNet-121 model achieved a test accuracy of 96.60% and a macro-averaged F1-score of 0.9655, with a training time of 39 min 44.41 s. Class-specific performance is presented in Table 2. The IK class was classified without error, with precision, recall, and F1-score values of 1.0000. The lowest class-specific recall was observed for HE (0.9100), whereas IA had the lowest precision (0.9238), indicating that most remaining errors involved the HE and IA classes.

Following the addition of the external SE block, DenseNet-121 achieved a test accuracy of 98.00% and a macro-averaged F1-score of 0.9781, with a training time of 40 min 42.16 s. The improvement was also evident at the class level: HE recall increased from 0.9100 to 0.9400, IA precision increased from 0.9238 to 0.9423, and IA recall increased from 0.9700 to 0.9800. The F1-score of the NS class increased from 0.9677 to 0.9875, while the IK class retained perfect classification performance. The class-specific results are presented in Table 3.

Compared with the standard model, external SE integration increased DenseNet-121 accuracy by 1.40 percentage points and the macro-averaged F1-score by 0.0126, while increasing the training time by 57.75 s. The standard ResNet-50 model achieved a test accuracy of 96.40% and a macro-averaged F1-score of 0.9627, with a training time of 39 min 27.19 s. Class-specific performance is presented in Table 4. The IK class was classified without error, with precision, recall, and F1-score values of 1.0000. The lowest F1-score was observed for IA (0.9327), followed by HE (0.9436).

Following the addition of the external SE block, ResNet-50 achieved a test accuracy of 97.20% and a macro-averaged F1-score of 0.9709, with a training time of 40 min 18.51 s. IA recall increased from 0.9700 to 0.9900, while its F1-score increased from 0.9327 to 0.9474. For the HE class, precision increased from 0.9684 to 0.9894 and the F1-score increased from 0.9436 to 0.9588. The IK class retained perfect classification performance. The class-specific results are presented in Table 5.

Compared with the standard model, external SE integration increased ResNet-50 accuracy by 0.80 percentage points and the macro-averaged F1-score by 0.0082, while increasing the training time by 51.32 s. The standard EfficientNet-B3 model achieved a test accuracy of 96.20% and a macro-averaged F1-score of 0.9594, with a training time of 1 h 6 min 3.60 s. Class-specific performance is presented in Table 6. The IK class was classified without error, with precision, recall, and F1-score values of 1.0000. The lowest F1-score was observed for HE (0.9246), followed by IA (0.9406). 

Following the addition of the external SE block, EfficientNet-B3 achieved a test accuracy of 96.80% and a macro-averaged F1-score of 0.9662, with a training time of 1 h 6 min 59.39 s. The most pronounced class-specific improvement was observed for HE: precision increased from 0.9293 to 0.9892 and the F1-score increased from 0.9246 to 0.9534, while recall remained unchanged at 0.9200. For IA, recall increased from 0.9500 to 0.9600, although precision decreased from 0.9314 to 0.9231, resulting in only a marginal change in F1-score from 0.9406 to 0.9412. The IK class retained a recall of 1.0000, while its precision decreased slightly to 0.9901. The class-specific results are presented in Table 7.

**Figure 4:**
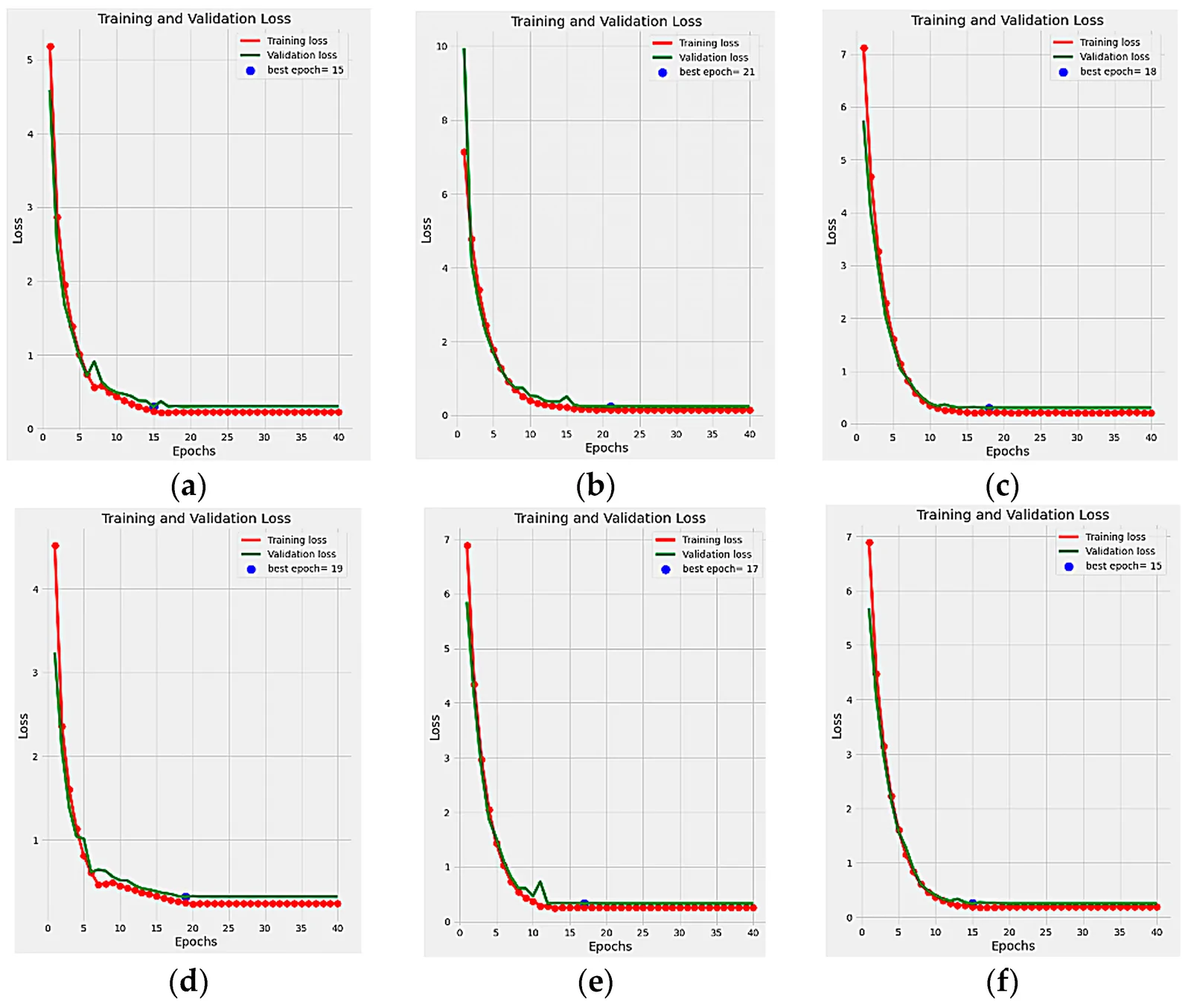
Training and validation loss curves of the evaluated CNN models: (**a**) standard DenseNet-121; (**b**) standard ResNet-50; (**c**) standard EfficientNet-B3; (**d**) SE-augmented DenseNet-121; (**e**) SE-augmented ResNet-50; and (**f**) EfficientNet-B3 with the additional external SE block.

Compared with the standard model, the additional external SE block increased EfficientNet-B3 accuracy by 0.60 percentage points and the macro-averaged F1-score by 0.0068, while increasing the training time by 55.79 s. The loss curves followed a similar pattern across the six model configurations in Figure 4. In each case, most of the decrease occurred during the first 10 epochs. The curves then changed more slowly and became stable toward the end of training. Training and validation loss remained close in the later epochs, and the external SE block did not noticeably alter the general convergence pattern.

**Figure 5:**
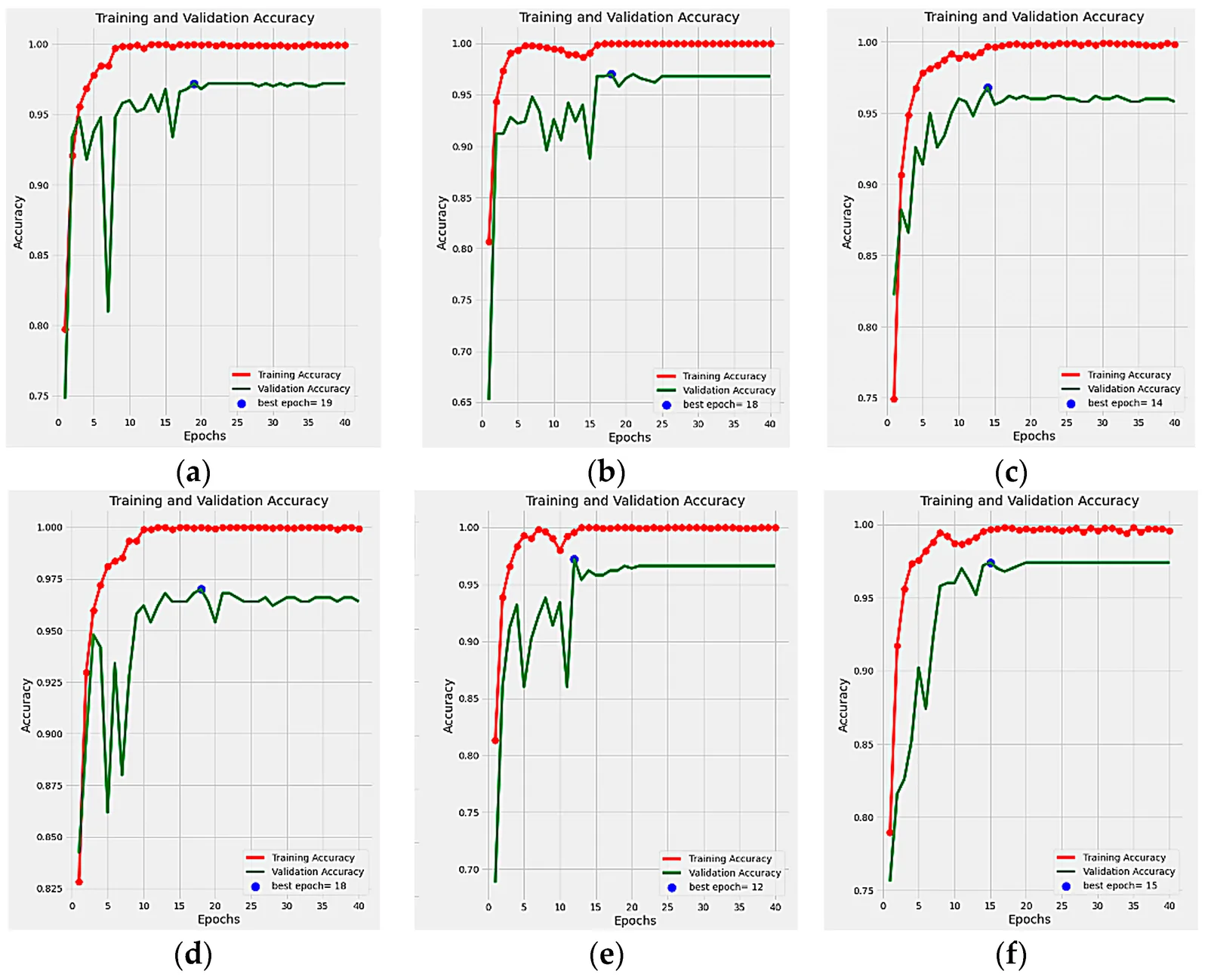
Training and validation accuracy curves of the evaluated CNN models: (**a**) standard DenseNet-121; (**b**) standard ResNet-50; (**c**) standard EfficientNet-B3; (**d**) SE-augmented DenseNet-121; (**e**) SE-augmented ResNet-50; and (**f**) EfficientNet-B3 with the additional external SE block.

A similar pattern can be seen in the accuracy curves in Figure 5. Training accuracy approached 1.00 in every configuration, while validation accuracy settled at a slightly lower level. The validation curves of DenseNet-121 and ResNet-50 fluctuated during the early and middle epochs, but these changes became smaller later in training. Since a small gap remained between training and validation accuracy, the final model comparison was based on the held-out test set.

**Figure 6:**
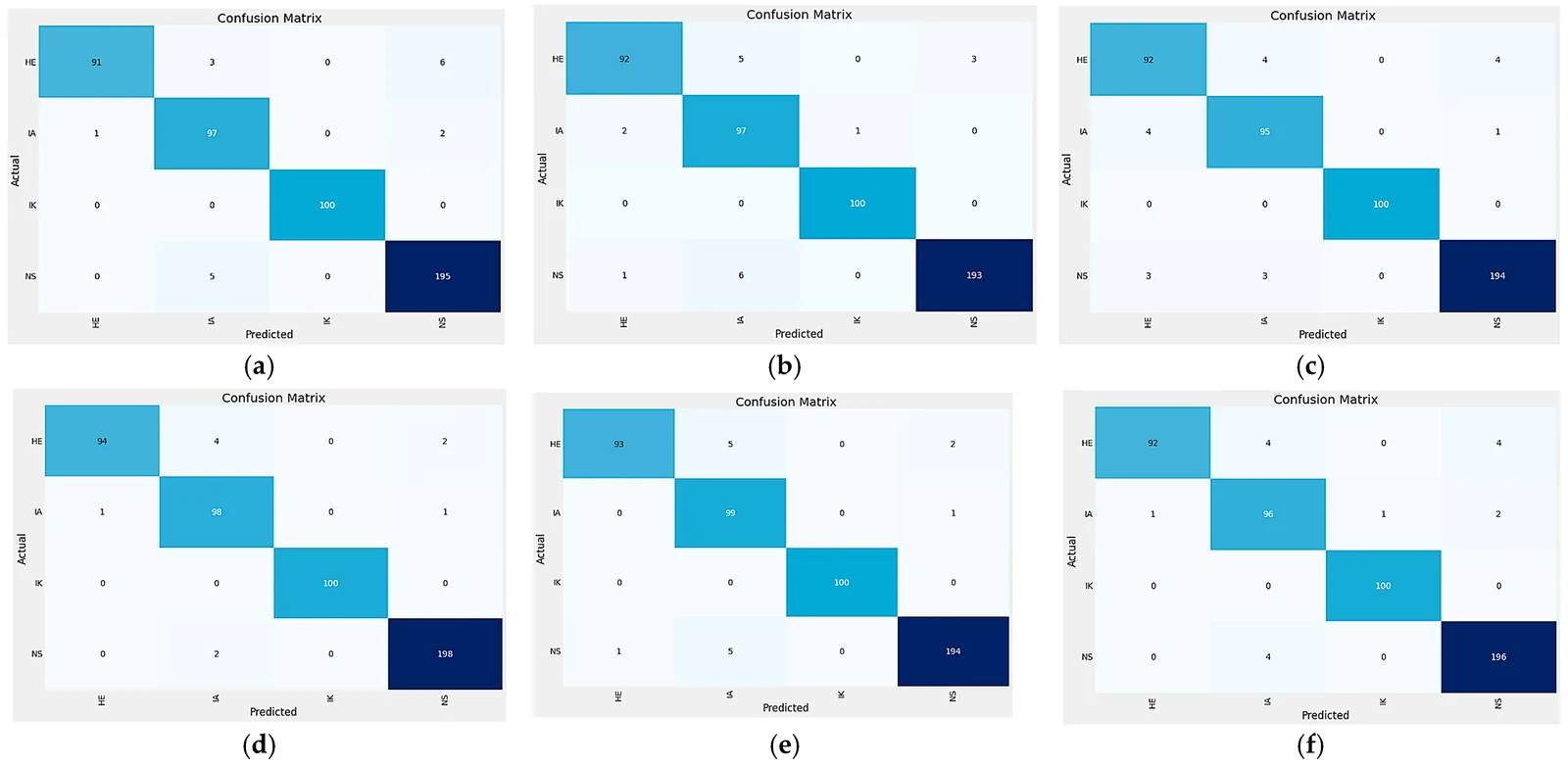
Test-set confusion matrices of the evaluated CNN models: (**a**) standard DenseNet-121; (**b**) standard ResNet-50; (**c**) standard EfficientNet-B3; (**d**) SE-augmented DenseNet-121; (**e**) SE-augmented ResNet-50; and (**f**) EfficientNet-B3 with the additional external SE block.

The confusion matrices show more clearly where the remaining classification errors occurred in Figure 6. All 100 IK images in the test set were correctly classified in every configuration. The other errors mainly involved the HE, IA, and NS classes. After the external SE block was added, the number of misclassified images fell from 17 to 10 for DenseNet-121, from 18 to 14 for ResNet-50, and from 19 to 16 for EfficientNet-B3. The largest reduction was therefore obtained with DenseNet-121, which also achieved the highest test accuracy.

External SE integration produced an improvement in test accuracy and macro-averaged F1-score for all three CNN backbones. The highest overall performance was obtained with SE-augmented DenseNet-121, which achieved an accuracy of 98.00% and a macro-averaged F1-score of 0.9781. DenseNet-121 also showed the largest accuracy gain following external SE integration (+1.40 percentage points), followed by ResNet-50 (+0.80 percentage points) and EfficientNet-B3 (+0.60 percentage points). The mean accuracy improvement across the three architectures was 0.93 percentage points. The corresponding increases in macro-averaged F1-score were 0.0126, 0.0082, and 0.0068, respectively. In all three architectures, the increase in training time remained below one minute. The complete comparison is presented in Table 8.

## 4. Discussion

In this study, integrating the Squeeze-and-Excitation (SE-Attention) channel attention mechanism into the DenseNet-121, ResNet-50, and EfficientNet-B3-based deep learning architectures used for classifying the pathological type of stroke (ischemic/hemorrhagic) and, in ischemic cases, its stage (acute/chronic) from brain CT images was found to consistently increase classification accuracy across all three architectures, with the most pronounced contribution observed in the DenseNet-121 architecture, in which accuracy increased from 96.60% to 98.00% (+1.40 points). SE-Attention integration produced notable improvements in the precision of the acute ischemic stroke (IA) class in particular, a class that is clinically difficult to distinguish, while imposing a negligible additional burden on model complexity and training time. These findings indicate that channel-based attention mechanisms can improve the diagnostic reliability of CT-based stroke classification systems at limited additional computational cost. Accordingly, the reported accuracy and macro-averaged F1-score differences should be interpreted as descriptive observations rather than evidence of statistical superiority.

From a biomimetic perspective, channel-wise recalibration provides a functional abstraction of selective information processing. Biological attention prioritizes task-relevant signals while reducing the influence of competing information [12]; similarly, the SE block strengthens feature channels that are more informative for classification and attenuates less useful responses [13]. The analogy concerns this shared principle of selective prioritization rather than the anatomical or physiological organization of biological attention. In contrast to a purely engineering-centered interpretation, which considers channel weighting mainly as a computational optimization operation, the biomimetic interpretation emphasizes how relevant information is selectively promoted within a larger set of competing feature responses.

Even the standard version of the DenseNet-121 architecture exhibited the highest baseline performance, with 96.60% accuracy and a macro F1-score of 0.9655, compared with the other two backbones (ResNet-50: 96.40%; EfficientNet-B3: 96.20%). This can be explained by the fact that the dense connectivity structure of the DenseNet architecture strengthens gradient flow by directly propagating the feature maps of earlier layers to later layers and increases feature reuse [14]. With SE-Attention integration, the accuracy of DenseNet-121 rose to 98.00% and its macro F1-score rose to 0.9781, the largest improvement observed among the three architectures considered. Similarly, the literature has reported that integrating channel attention mechanisms into densely connected architectures helps highlight discriminative features by reducing redundant information between channels [20,21].

In the ResNet-50 architecture, SE-Attention integration increased accuracy from 96.40% to 97.20% and the macro F1-score from 0.9627 to 0.9709. This finding is consistent with the substantial performance gain reported by Umapathy et al. [22] using an SE-ResNeXt-based ensemble architecture for classifying intracranial hemorrhage subtypes, and with the improvement findings reported by Xu et al. [23] using SE-based architectures for analyzing hemorrhagic stroke CT images. It is thought that when the channel recalibration provided by the SE block is added to the stable gradient flow provided by skip connections, an additional contribution is made to distinguishing the low-contrast tissues characteristic of the hemorrhage region.

For EfficientNet-B3, the comparison should be interpreted as an assessment of the incremental contribution of an additional external SE block rather than the introduction of SE attention into an otherwise SE-free architecture. In the EfficientNet-B3 architecture, the effect of SE-Attention integration was more limited compared with the other two architectures (96.20% to 96.80%), which can be explained by the fact that, within the compound scaling approach of the EfficientNet family, the MBConv blocks already contain an SE module [15] consequently, the marginal contribution of a second, externally added SE block was lower than for the DenseNet-121 and ResNet-50 backbones, which entirely lack an SE mechanism. A similar observation was reported in a multi-class stroke classification study conducted by Kulathilake et al. [24] using an extended ResNet architecture, in which the contribution of additional modules was reduced when attention-like recalibration mechanisms were already part of the backbone.

When evaluated on a per-class basis, the chronic ischemic stroke (IK) class was distinguished with near-perfect accuracy (precision/recall ≈ 1.00) both before and after SE-Attention across all three architectures, whereas the acute ischemic stroke (IA) class had the relatively lowest precision values across all models. However, this finding is specific to the present dataset and labeling scheme and should not be interpreted as evidence of clinically reliable recognition of chronic ischemic lesions in broader patient populations. This is consistent with the fact that acute ischemic stroke findings appear on CT images in the early period as low-contrast and poorly defined findings [3,4,10]. SE-Attention integration increased the precision of the IA class from 0.9238 to 0.9423 for DenseNet-121 and from 0.8981 to 0.9083 for ResNet-50; this finding is consistent with the literature indicating that channel attention mechanisms strengthen the representation of low-contrast acute ischemic lesions by suppressing non-discriminative, noisy channels [20,21]. The consistent rise of precision values above 98% for the hemorrhagic stroke (HE) class after SE-Attention indicates that the attention mechanism more effectively highlights the hyperdense areas characteristic of hemorrhage regions [22,23].

The accuracy rates obtained are also competitive when compared with previous studies targeting a single subtask (either only hemorrhagic/ischemic differentiation or only ICH subtype classification). Chin et al. [6] reported 84% accuracy on a single binary classification problem, while Alhatemi and Savaş [9] achieved 98.80% accuracy on MRI images; however, most of these studies were conducted on single-center datasets with fewer classes. In contrast, the present study combines pathological type and stage information within a single framework using 5000 images obtained from two different centers in a four-class (NS, HE, IA, IK), multicenter design; in this respect, it presents a multi-task classification scenario closer to clinical workflow, similar to the OzNet-based study of Ozaltin et al. [25] and the explainable CNN approach of Abdi et al. [26]. Furthermore, the high-sensitivity findings obtained by UmaMaheswaran et al. [27] using a machine learning approach based on hybrid preprocessing and feature selection also support the notion that the differentiation of early findings on non-contrast CT images can be substantially improved through appropriate feature-highlighting strategies.

The findings summarized in Table 8 show that external SE integration consistently improved test accuracy, while the increase in training time remained below approximately one minute for each architecture, which is consistent with the computational-efficiency findings reported in the literature for MedNet [28] and similar lightweight attention modules. Nevertheless, some limitations of the study should be discussed. First, the dataset is limited to 5000 CT images obtained from two centers (Elazığ Fethi Sekin City Hospital and Dicle University Hospital); this may limit the generalizability of the model to images obtained with different CT devices and imaging protocols [24,26]. Second, the present study does not employ Grad-CAM-like explainable artificial intelligence (XAI) techniques that would increase the transparency of the model’s decision-making process, whereas the current literature emphasizes that such visualization methods are of critical importance for clinical decision-support systems to be reliably adopted by radiologists [22,29,30]. Third, equal class representation during model optimization was achieved by subsampling the NS class and augmenting the stroke-related classes; therefore, this balanced training distribution may not reflect class frequencies encountered in routine clinical practice [27,31]. 

Finally, only the SE block was evaluated in this study; no comparative analysis was performed with alternative mechanisms, such as CBAM, that jointly model both channel and spatial attention [31]. For future studies, external validation on multicenter and larger-scale datasets, integration of XAI-based visualizations into the system, and comparison of the SE block with hybrid attention architectures such as CBAM are recommended [32,33]. Overall, the findings indicate that the SE-Attention mechanism provided descriptive improvements in internal test-set classification performance at low additional computational cost. These results support technical feasibility within the present dataset but do not establish diagnostic value or clinical readiness.

### Limitations of the Study and Future Work

This study has several limitations. First, only three different convolutional neural network (CNN) architectures were evaluated: DenseNet-121, ResNet-50, and EfficientNet-B3. Although these architectures demonstrated high classification performance, more recent deep learning architectures developed in recent years, such as Vision Transformer (ViT), Swin Transformer, and CNN–Transformer hybrid models, were not included in the study. Evaluating these architectures in future studies would allow a more comprehensive comparison of the performance of the proposed approach.

Second, the developed models were trained and evaluated using only brain CT images. Integrating additional clinical data, such as clinical history, age, sex, laboratory parameters, and neurological examination findings, together with imaging data into multimodal artificial intelligence models could both improve classification success and strengthen the model’s clinical applicability.

Finally, although the contribution of the SE-Attention mechanism to classification performance was evaluated in this study, the interpretability of the model’s decisions was not examined. Using Grad-CAM, Score-CAM, and similar Explainable Artificial Intelligence (XAI) methods in future studies to visualize which anatomical regions the models rely on during decision-making would increase the transparency and clinical reliability of the model.

## 5. Conclusions

In this study, a channel-based Squeeze-and-Excitation (SE-Attention) attention mechanism was integrated into DenseNet-121-, ResNet-50-, and EfficientNet-B3-based deep learning architectures for multi-class brain stroke classification from brain CT images, and the contribution of this approach to classification performance was comprehensively evaluated. The findings obtained demonstrate that the SE-Attention mechanism enables more effective learning of discriminative features across all CNN-based architectures, contributing to higher classification success compared with the base models.

Indeed, the SE-Attention mechanism could be easily integrated into the proposed hybrid CNN-based architectures without introducing a significant computational burden or cost. Although the effect of this integration on the architectures considered was not uniform, an average accuracy improvement of 0.93% was achieved in the classification tasks, with this improvement exceeding 1% for some architectures.

One of the most important aspects of this study is that it does not focus solely on determining the pathological type of stroke (ischemic/hemorrhagic) but is also able to distinguish between acute and chronic ischemic stroke, which is of critical importance in the clinical decision-making process. This two-stage approach increases the clinical usability potential of decision-support systems in time-critical stroke management in emergency departments. In addition, the use of a two-center dataset labeled by radiology specialists provided a common basis for comparing the evaluated model configurations.

The performance improvement provided by the SE-Attention mechanism demonstrates that channel-based feature reweighting enables more effective representation of subtle radiological findings in brain CT images. This highlights the importance of attention mechanisms in learning low-contrast, difficult-to-distinguish lesions such as early-stage ischemic changes. The present findings support the technical feasibility of attention-based CNN architectures under the reported experimental setting, but patient-disjoint external validation is required before their clinical use can be considered.

## Figures and Tables

**Table 1:** Image counts resulting from image-level partitioning and training-set balancing in StrokeCT-2C5K.

Class	Original Dataset	Training Before Balancing	Training After Balancing	Validation	Test
Normal (NS)	2000	1600	1000	200	200
Hemorrhagic stroke (HE)	1000	800	1000	100	100
Acute ischemic stroke (IA)	1000	800	1000	100	100
Chronic ischemic stroke (IK)	1000	800	1000	100	100
Total	5000	4000	4000	500	500

**Table 2:** Class-specific test performance of the standard DenseNet-121 model.

Classification Report: DenseNet-121 (Standard)				
	Precision	Recall	F1-Score	Support
**HE**	0.9891	0.9100	0.9479	100
**IA**	0.9238	0.9700	0.9463	100
**IK**	1.0000	1.0000	1.0000	100
**NS**	0.9606	0.9750	0.9677	200

**Table 3:** Class-specific test performance of the SE-augmented DenseNet-121 model.

Classification Report: DenseNet-121 + SE-Attention				
Class	Precision	Recall	F1-Score	Support
**HE**	0.9895	0.9400	0.9641	100
**IA**	0.9423	0.9800	0.9608	100
**IK**	1.0000	1.0000	1.0000	100
**NS**	0.9851	0.9900	0.9875	200

**Table 4:** Class-specific test performance of the standard ResNet-50 model.

Classification Report: ResNet-50 (Standard)				
Class	Precision	Recall	F1-Score	Support
**HE**	0.9684	0.9200	0.9436	100
**IA**	0.8981	0.9700	0.9327	100
**IK**	1.0000	1.0000	1.0000	100
**NS**	0.9847	0.9650	0.9747	200

**Table 5:** Class-specific test performance of the SE-augmented ResNet-50 model.

Classification Report: ResNet-50 + SE-Attention				
Class	Precision	Recall	F1-Score	Support
**HE**	0.9894	0.9300	0.9588	100
**IA**	0.9083	0.9900	0.9474	100
**IK**	1.0000	1.0000	1.0000	100
**NS**	0.9848	0.9700	0.9773	200

**Table 6:** Class-specific test performance of the standard EfficientNet-B3 model.

Classification Report: EfficientNet-B3 (Standard)				
Class	Precision	Recall	F1-Score	Support
**HE**	0.9293	0.9200	0.9246	100
**IA**	0.9314	0.9500	0.9406	100
**IK**	1.0000	1.0000	1.0000	100
**NS**	0.9749	0.9700	0.9724	200

**Table 7:** Class-specific test performance of the EfficientNet-B3 model with the additional external SE block.

Classification Report: EfficientNet-B3 + SE-Attention				
Class	Precision	Recall	F1-Score	Support
**HE**	0.9892	0.9200	0.9534	100
**IA**	0.9231	0.9600	0.9412	100
**IK**	0.9901	1.0000	0.9950	100
**NS**	0.9703	0.9800	0.9751	200

**Table 8:** Overall comparison of baseline and external SE-augmented models on the test set.

Backbone	Configuration	Accuracy (%)	Macro-Averaged F1-Score	Training Time	Accuracy Gain (pp)
DenseNet-121	Standard	96.6	0.9655	39 min 44.41 s	—
DenseNet-121	External SE	98	0.9781	40 min 42.16 s	1.4
ResNet-50	Standard	96.4	0.9627	39 min 27.19 s	—
ResNet-50	External SE	97.2	0.9709	40 min 18.51 s	0.8
EfficientNet-B3	Standard	96.2	0.9594	1 h 6 min 3.60 s	—
EfficientNet-B3	Additional external SE	96.8	0.9662	1 h 6 min 59.39 s	0.6

SE, squeeze and excitation; pp, percentage points. Accuracy gain was calculated relative to the corresponding baseline model.

## Data Availability

The StrokeCT-2C5K dataset generated and analyzed in this study will be made publicly available through a GitHub repository upon acceptance of the manuscript.
